# Valley District Dental Society, Massachusetts—Resolutions of Respect

**Published:** 1903-01

**Authors:** 


### Obituary.

### VALLEY DISTRICT DENTAL SOCIETY, MASSACHUSETTS—RESOLUTIONS OF RESPECT.

It is with deep regret that this Society learns of the death of our esteemed fellow-member, Dr. J. Searle Hurlbut, of Springfield, on November 9, 1902, and we desire to express in a formal manner our appreciation of his life and a sense of the loss we have sustained.

Dr. Hurlbut commenced the practice of dentistry in Springfield in 1865, having graduated from the Philadelphia Dental College that year, and during all these years has stood firm for the up-building of his profession. He was a member of the old Connecticut Valley Dental Society—the leading dental society in New England—from 1865 until 1895, when it was merged into the Northeastern Dental Association, of which his membership continued until his death. He became a member of the Massachusetts Dental Society in 1873, and was thus one of the original members of this the Valley District Dental Society. He was also a member of the American Academy of Dental Science, of Boston, of the Odontological Society of New York and of the National Dental Association. He was honored as president by the Connecticut Valley Dental Society in 1873, and by the Massachusetts Dental Society in 1874. When the dental law was enacted in this State, in 1887, he was appointed by Governor Ames a member of the Board of Registration in Dentistry. In 1891 he was made president of this board, which office he held until he resigned his membership in 1896.

By his strong personality, his broadly cultivated views, his refined manner, and dignified bearing he was one of the leaders in influencing an intelligent public to that just appreciation of the dental profession which later years have witnessed.

*Resolved,* That in the death of Dr. J. Searle Hurlbut the members of this Society feel they have sustained a personal loss and the dental profession one of its eminent members.

*Resolved,* That we extend to his wife our most sincere and heartfelt sympathy.

*Resolved,* That a copy of these resolutions be sent to his wife, to the various dental journals, and to the daily papers.

George A. Maxfield,
C. T. Stockwell,
N. Morgan,
*Committee.*

